# Cardiac [^99m^Tc]Tc-hydroxydiphosphonate uptake on bone scintigraphy in patients with hereditary transthyretin amyloidosis: an early follow-up marker?

**DOI:** 10.1007/s00259-023-06459-y

**Published:** 2023-10-16

**Authors:** H. S. A. Tingen, A. Tubben, J. Bijzet, M. P. van den Berg, P. van der Meer, E. J. Houwerzijl, F. L. H. Muntinghe, P. A. van der Zwaag, A. W. J. M. Glaudemans, M. I. F. J. Oerlemans, C. Knackstedt, M. Michels, A. Hirsch, B. P. C. Hazenberg, R. H. J. A. Slart, H. L. A. Nienhuis

**Affiliations:** 1 Department of Nuclear Medicine and Molecular Imaging, University Medical Centre Groningen and Amyloidosis Centre of Expertise, Groningen, The Netherlands; 2 Department of Cardiology, University Medical Centre Groningen and Amyloidosis Centre of Expertise, Groningen, The Netherlands; 3 Department of Rheumatology & Clinical Immunology, University Medical Centre Groningen and Amyloidosis Centre of Expertise, Groningen, The Netherlands; 4 Department of Internal Medicine, University Medical Centre Groningen and Amyloidosis Centre of Expertise, Groningen, The Netherlands; 5 Department of Clinical Genetics, University Medical Centre Groningen and Amyloidosis Centre of Expertise, Groningen, The Netherlands; 6 Department of Cardiology and Member of the European Reference Network for rare, low prevalence and complex diseases of the heart: ERN GUARD-Heart , University Medical Centre Utrecht, Utrecht, The Netherlands; 7 Department of Cardiology, Cardiovascular Research Institute Maastricht (CARIM), Maastricht University Medical Centre +, Maastricht, The Netherlands; 8 Department of Cardiology, University Medical Centre Rotterdam, Rotterdam, The Netherlands; 9 Department of Radiology and Nuclear Medicine, University Medical Centre Rotterdam, Rotterdam, The Netherlands; 10 Biomedical Photonic Imaging Group, Faculty of Science and Technology, University of Twente, Enschede, The Netherlands

**Keywords:** ATTR-CM, ATTRv amyloidosis, Bone scintigraphy, Gene silencing, TTR stabilizers, Monitoring

## Abstract

**Purpose:**

There is a need for early quantitative markers of potential treatment response in patients with hereditary transthyretin (ATTRv) amyloidosis to guide therapy. This study aims to evaluate changes in cardiac tracer uptake on bone scintigraphy in ATTRv amyloidosis patients on different treatments.

**Methods:**

In this retrospective cohort study, outcomes of 20 patients treated with the transthyretin (*TTR)* gene silencer patisiran were compared to 12 patients treated with a TTR-stabilizer. Changes in NYHA class, cardiac biomarkers in serum, wall thickness, and diastolic parameters on echocardiography and NYHA class during treatment were evaluated.

**Results:**

Median heart/whole-body (H/WB) ratio on bone scintigraphy decreased from 4.84 [4.00 to 5.31] to 4.16 [3.66 to 4.81] (*p* < .001) in patients treated with patisiran for 29 [15–34] months. No changes in the other follow-up parameters were observed. In patients treated with a TTR-stabilizer for 24 [20 to 30] months, H/WB ratio increased from 4.46 [3.24 to 5.13] to 4.96 [ 3.39 to 5.80] (*p* = .010), and troponin T increased from 19.5 [9.3 to 34.0] ng/L to 20.0 [11.8 to 47.8] ng/L (*p* = .025). All other parameters did not change during treatment with a TTR-stabilizer.

**Conclusion:**

A change in cardiac tracer uptake on bone scintigraphy may be an early marker of treatment-specific response or disease progression in ATTRv amyloidosis patients.

**Supplementary Information:**

The online version contains supplementary material available at 10.1007/s00259-023-06459-y.

## Introduction

Hereditary transthyretin (ATTRv) amyloidosis is a progressive disease characterized by the extracellular deposition of transthyretin (TTR)-derived amyloid fibrils in various organs and tissues. The heart and nervous system are most frequently affected [1], and cardiac involvement is associated with progressive heart failure and reduced survival [2]. Non-invasive diagnosis of ATTR-related cardiomyopathy (ATTR-CM) is based on bone scintigraphy findings, which has a very high accuracy to detect ATTR-CM provided that light chain amyloidosis has been ruled out [3, 4].

Nowadays, several treatment options are available for patients suffering from ATTRv amyloidosis [1]. TTR-stabilizers (tafamidis and diflunisal) have been studied and used for over a decade and have proven to retard disease progression and improve outcomes in patients with ATTR-CM [5–7] and polyneuropathy [7–9]. Recently, *TTR* gene silencing has been shown to halt or even reduce neuropathy and improve cardiac parameters in ATTR amyloidosis patients [10–15].

As multiple treatment options are available now, there is a need for early and quantitative markers of disease progression and treatment response, allowing to make early treatment decisions and to apply the new treatments as effectively as possible. The primary objective of this study is to evaluate the utility of [^99m^Tc]Tc-hydroxydiphosphonate (HDP) bone scintigraphy in analyzing treatment efficacy in ATTRv amyloidosis patients treated with patisiran and TTR-stabilizers.

## Methods

### Study population

In this retrospective cohort study, ATTRv amyloidosis patients with cardiomyopathy treated with patisiran at the National Amyloidosis Centre of Expertise of the University Medical Centre Groningen in The Netherlands, were enrolled. All patients with a baseline bone scintigraphy and at least one follow-up bone scintigraphy were included. Baseline bone scintigraphy had to be performed less than 9 months before or within 2 months after start of patisiran. Follow-up bone scintigraphy had to be performed at least 11 months after the start of patisiran. If present, a subsequent bone scintigraphy was also included as additional follow-up scan.

Additionally, from a historical cohort, ATTRv amyloidosis patients treated with a TTR-stabilizer were included if two bone scintigraphies were performed during treatment. Twelve patients with a similar follow-up duration as the patisiran group were selected.

NYHA class, results of blood tests, and echocardiograms were retrieved from the medical records. Only tests performed within 6 months before and less than 6 months after bone scintigraphies were selected.

Diagnosis of ATTRv amyloidosis was based on the presence of a pathogenic variant in the *TTR* gene in combination with biopsy-proven amyloid. Diagnosis of ATTR-CM was based on the diagnostic algorithm described by Garcia-Pavia [3].

All procedures were in compliance with the Declaration of Helsinki. The study was approved by the institutional review board of the UMCG, and requirement for consent was waived (Registration number: 202100406).

### Cardiac biomarkers

NT-proBNP and troponin T were measured as part of routine clinical care. All values during treatment were extracted from the patient files.

### Echocardiography

All echocardiograms were reviewed by cardiologists as part of routine clinical care and according to clinical standards [16]. Interventricular septal wall thickness (IVSt), left ventricular posterior wall thickness (LVPWt), E′mean (average calculated from E′lateral and E′septal), E/e′ ratio, E/A ratio, and mitral valve deceleration time (MVDT) were extracted from the medical records. Data on global longitudinal strain (GLS) were not available in the majority of patients and were therefore not included in the analysis.

### Bone scintigraphy

All patients received 450–750 MBq [^99m^Tc]Tc-HDP intravenously for each of both scans. Bone scintigraphies were performed on dedicated SPECT/CT systems (Symbia T2, Symbia T16 and Symbia Intevo, Siemens Healthineers, Erlangen, Germany) equipped with a low-energy high-resolution collimator. Planar whole-body scans were obtained 3 h after injection from posterior and anterior view. Acquisition time was 12 min in total, and 6 min per view. SPECT images were acquired using a 180° configuration, 64 views, 20 s per view and a 128 × 128 matrix. Planar images were compared with single-photon emission computed tomography (SPECT) images to rule out blood pool activity. Anterior planar images were scored according to the Perugini scale [17], and a free-handed region of interest (ROI) was placed over the heart, oval ROIs were placed over the kidneys, and bladder and a free-handed ROI was placed around the outline of the body. Heart/whole-body (H/WB) ratio was calculated [18].

### Statistics

Results are presented as median [interquartile range]. To evaluate change over time, values at baseline and during follow-up were compared using the Wilcoxon matched-pair signed-rank test.

To compare the change in parameters between both treatment groups, the baseline value of the parameters was subtracted from the value during treatment. If multiple follow-up tests were available, the tests with the longest follow-up were used. The resulting value represented the change in parameter during treatment. Continuous variables were compared using a Mann–Whitney *U* test, and ordinal variables were compared using a Fisher’s exact test.

The level of significance for all hypothesis tests was set at 0.05. All data were analyzed using SPSS version 26 software (IBM Corp, Armonk, New York, USA).

## Results

### Clinical and demographic characteristics

Two groups were analyzed in this study; one group treated with patisiran (with or without additional treatment with a TTR-stabilizer) and one group treated with a TTR-stabilizer only.

In the patisiran group, 20 patients were included and received 0.3 mg/kg patisiran intravenously every 3 weeks for at least 11 months for the indication of polyneuropathy. Eight of these patients (40%) received additional treatment with tafamidis (seven patients received 20 mg and one patient received 80 mg daily), and three patients received additional treatment with diflunisal (twice daily 250 mg). These patients were already on treatment with a TTR-stabilizer before the start of patisiran, and the physician decided to continue this treatment next to treatment with patisiran. Five patients (25%) were treatment naïve at the start of patisiran, and in seven (35%) patients, treatment with a TTR-stabilizer was discontinued upon initiation of patisiran. At baseline, five patients (25%) were treatment naïve.

In the TTR-stabilizer only group, 12 patients were included of which nine patients were treated with tafamidis (all patients received 20 mg daily) and three patients were treated with diflunisal (twice daily 250 mg). At baseline, three patients (25%) were treatment naïve.

The median age of patients, sex distribution, time from diagnosis, follow-up time from baseline, number of treatment naïve patients, time between tracer administration and scan acquisition, total number of counts on the anterior planar view of bone scintigraphy, and H/WB ratio did not differ between the groups. IVSt, LVPWt, E′ mean, and E/e′ ratio were significantly higher in the patisiran group as compared to the stabilizer group (Table 1). Organ involvement did not differ between the groups. Genotype distribution and other characteristics are shown in Table 1.

**Table 1:** Patient characteristics at baseline

Characteristic	Group 1	Group 2	*P* value
Treatment	Patisiran^a^	TTR-stabilizer	
Number of patients	20	12	
Age (years)	67 [58–71]	67 [61–72]	ns
Male	16 (80%)	9 (75%)	n
Time from diagnosis (years)	3 [0–4]	2 [0–3]	ns
Follow-up (months)	29 [15–34]	24 [20–30]	ns
Treatment naive	5 (25%)	3 (25%)	ns
Time between tracer injection and bone scintigraphy (minutes)			
Baseline scan	180 [164–193]	180 [180–180]	ns
First follow-up scan	188 [167–200]	180 [180–180]	ns
Second follow-up scan	199 [175–216]	n/a	n/a
Total counts anterior planar bone scintigraphy (kCounts)			
Baseline scan	1233.31 [1046.19–1477.55]	14505.81 [1080.80–1810.41]	ns
First follow-up scan	1215.02 [997.49–1327.56]	1446.60 [1105.22–1578.48]	ns
Second follow-up scan	1070.77 [978.97–1380.43]	n/a	n/a
Serum biomarkers			
Troponin T (ng/L)	26.0 [10.0–39.3]	19.5 [9.3–34.0]	ns
NT-proBNP (ng/L)	418 [127–972]	315 [166–1242]	ns
eGFR (ml/min*1.73m^2^)	89 [75–94]	82 [69–94]	ns
Echocardiography			
IVSt (mm)	15 [13–20]	11 [9–11]	.002*
LVPWt (mm)	13 [12–14]	10 [9–11]	< .001*
E′ mean (cm/s)	6.4 [5.3–7.2]	8.0 [7.2–9.4]	.006*
E/e′ ratio	13.2 [10.1–18.4]	6.8 [6.0–8.0]	.009*
E/A ratio	0.9 [0.9–1.1]	0.8 [0.7–1.7]	ns
MVDT (ms)	210 [169–220]	248 [203–360]	ns
Bone scintigraphy			
H/WB ratio	4.84 [4.00–5.31]	4.46 [3.24–5.13]	ns
Perugini score:			ns
1	2 (10%)	4 (33%)	
2	7 (35%)	4 (33%)	
3	11 (55%)	4 (33%)	
NYHA class			ns
I	8 (40%)	9 (75%)	
II	6 (30%)	3 (25%)	
III	4 (20%)	0 (0%)	
IV	2 (10%)	0 (0%)	
Organ involvement			
Polyneuropathy	20 (100%)	12 (100%)	ns
Autonomic neuropathy	9 (45%)	7 (58%)	ns
Ocular involvement	4 (20%)	3 (25%)	ns
Genotype			ns
TTRV30M (p.Val50Met)	13 (65%)	8 (67%)	
TTRV71A (p.Val91Ala)	2 (10%)	1 (8%)	
TTRS23N (p.Ser43Asn)	2 (10%)	0 (0%)	
TTRE89K (p.Glu109Lys)	1 (5%)	1 (8%)	
TTRV94A (p.Val114Ala)	1 (5%)	0 (0%)	
TTRI107V (p.Ile127Val)	1 (5%)	0 (0%)	
TTRA45G (p.Ala65Gly)	0 (0%)	1 (8%)	
TTRH88R (p.His108Arg)	0 (0%)	1 (8%)	

Values are median [interquartile range] or number of patients (percentage)

*n/a*, not applicable; *NT-proBNP*, N-terminal brain natriuretic propeptide; *eGFR*, estimated glomerular filtration rate; *IVSt*, interventricular septal wall thickness; *LVPWt*, left ventricular posterior wall thickness; *MVDT*, mitral valve deceleration time; *H/WB ratio*, heart/whole-body ratio; *NYHA*, New York Heart Association classification of heart failure; * means significant difference; *ns*, not significant; *a*, eight patients additionally received a TTR-stabilizer

### Follow-up during treatment with patisiran

**Fig. 1:**
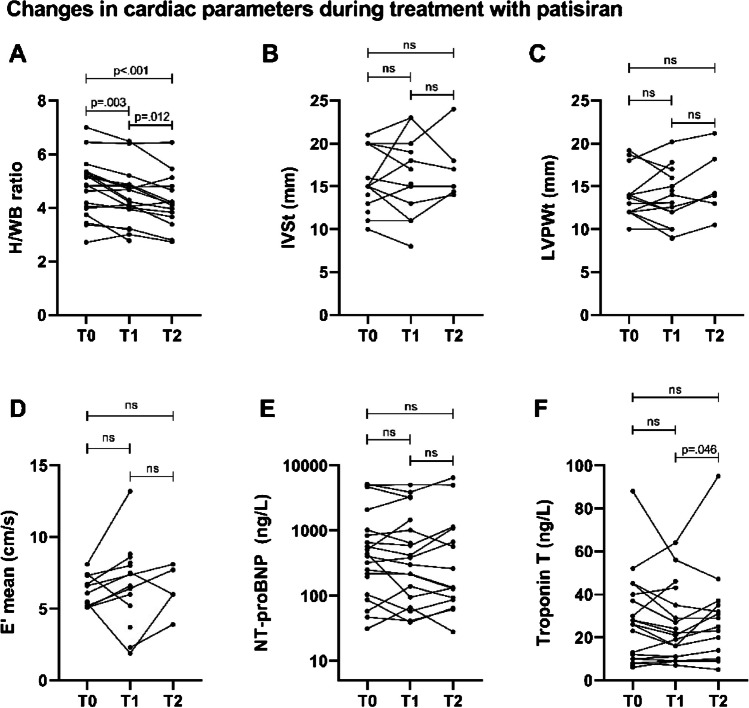
Change in cardiac parameters during treatment with patisiran. **A** H/WB ratio on bone scintigraphy. **B** IVSt on echocardiography. **C** LVPWt on echocardiography. **D** E′ mean on echocardiography. **E** NT-proBNP levels. **F** Troponin T levels. H/WB, heart/whole-body; IVSt, interventricular septal wall thickness; LVPWt, left ventricular posterior wall thickness; NT-proBNP, N-terminal brain natriuretic propeptide; ns, not significant

**Fig. 2:**
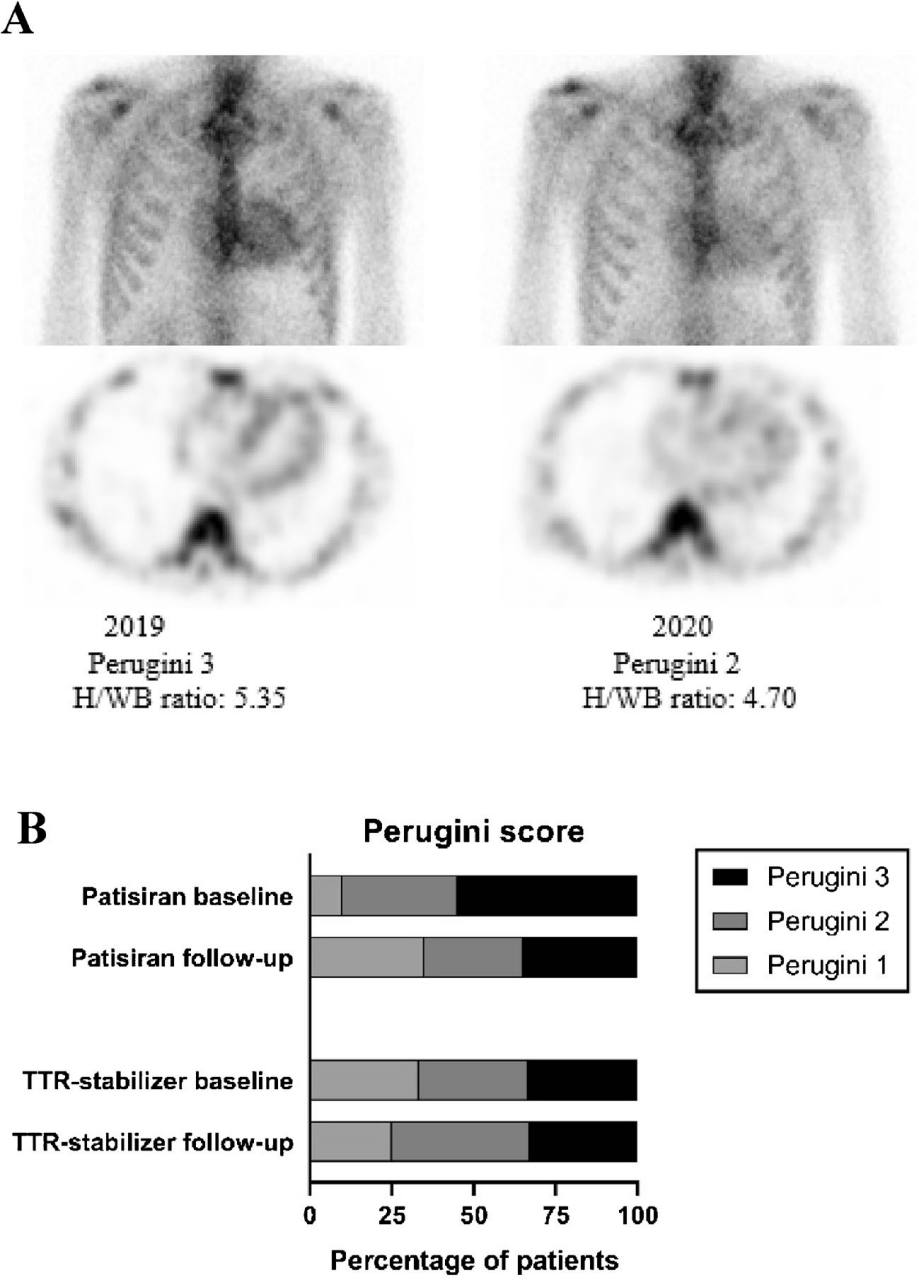
Change in cardiac tracer uptake on bone scintigraphy during treatment with patisiran. **A** Planar images (above) and transversal SPECT images (below) of bone scintigraphy in a patient before start of patisiran (left) and after 12 months of treatment (right). **B** Bar-chart of the distribution of Perugini score at baseline and during follow-up in both treatment groups. SPECT, single-photon emission computed tomography; H/WB, heart/whole body

During treatment with patisiran, adequate serum TTR reduction was achieved and median reduction was 81% [79 to 87%]. Median follow-up duration was 29 [15 to 34 months]. First bone scintigraphy during follow-up was performed at median 12 [11 to 14] months after the start of patisiran, and for 15 patients (75%), a second follow-up scan was available, performed median 30 [28 to 35] months after the start of patisiran. H/WB ratio decreased from median 4.84 [4.00 to 5.31] to median 4.49 [3.96 to 4.86] (*p* = 0.003) between baseline and the first follow-up scan and decreased further to 4.16 [3.66–4.81] (*p* = 0.012) in the 15 patients for whom a second follow-up scan was available (Fig. 1A). Three patients additionally showed a decrease in Perugini score of one point, and two patients showed a decrease of two points (*p* = 0.038) (Fig. 2). No significant changes in NYHA class, NT-proBNP, or echocardiographic parameters were observed during treatment with patisiran (Fig. 1B–E). An increase in troponin T from 21.0 [11.0–35.0] ng/L to 25.0 [14.0–35.0] ng/L (*p* = 0.046) was observed between the first and second follow-up assessment in the 15 patients with two follow-up bone scintigraphies. However, no significant change was observed between baseline and either of the follow-up scans (Fig. 1F).

Change in all cardiac follow-up parameters did not differ between patients treated with patisiran only and patients treated with both patisiran and a TTR-stabilizer (Online Resource 1).

### Follow-up during treatment with a TTR-stabilizer

**Fig. 3:**
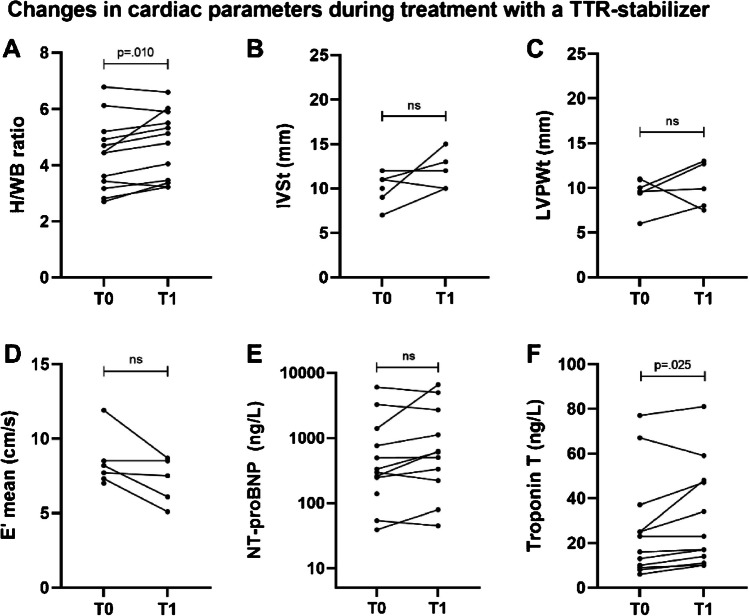
Change in cardiac parameters during treatment with a TTR-stabilizer. **A** H/WB ratio on bone scintigraphy. **B** IVSt on echocardiography. **C** LVPWt on echocardiography. **D** E′ mean on echocardiography. **E** NT-proBNP levels. **F** troponin T levels; TTR, transthyretin; H/WB, heart/whole-body; IVSt, interventricular septal wall thickness; LVPWt, left ventricular posterior wall thickness; NT-proBNP, N-terminal brain natriuretic propeptide; ns, not significant

Median follow-up was 24 [20 to 30] months in patients treated with a TTR-stabilizer. During treatment, an increase in H/WB ratio from a median of 4.46 [3.24 to 5.13] to 4.96 [ 3.39 to 5.80] (*p* = 0.010) was observed. Troponin T increased changed from a median of 19.5 [9.3 to 34.0] ng/L to 20.0 [11.8 to 47.8] ng/L (*p* = 0.025) (Fig. 3F). No significant changes in NYHA class, NT-proBNP levels, and echocardiographic parameters and Perugini score on bone scintigraphy were observed (Fig. 3B–E). In a subanalysis of patients treated with tafamidis (after exclusion of patients treated with diflunisal), no significant changes during treatment could be observed in any of the follow-up parameters (data not shown).

### Comparison between treatment groups

**Fig. 4:**
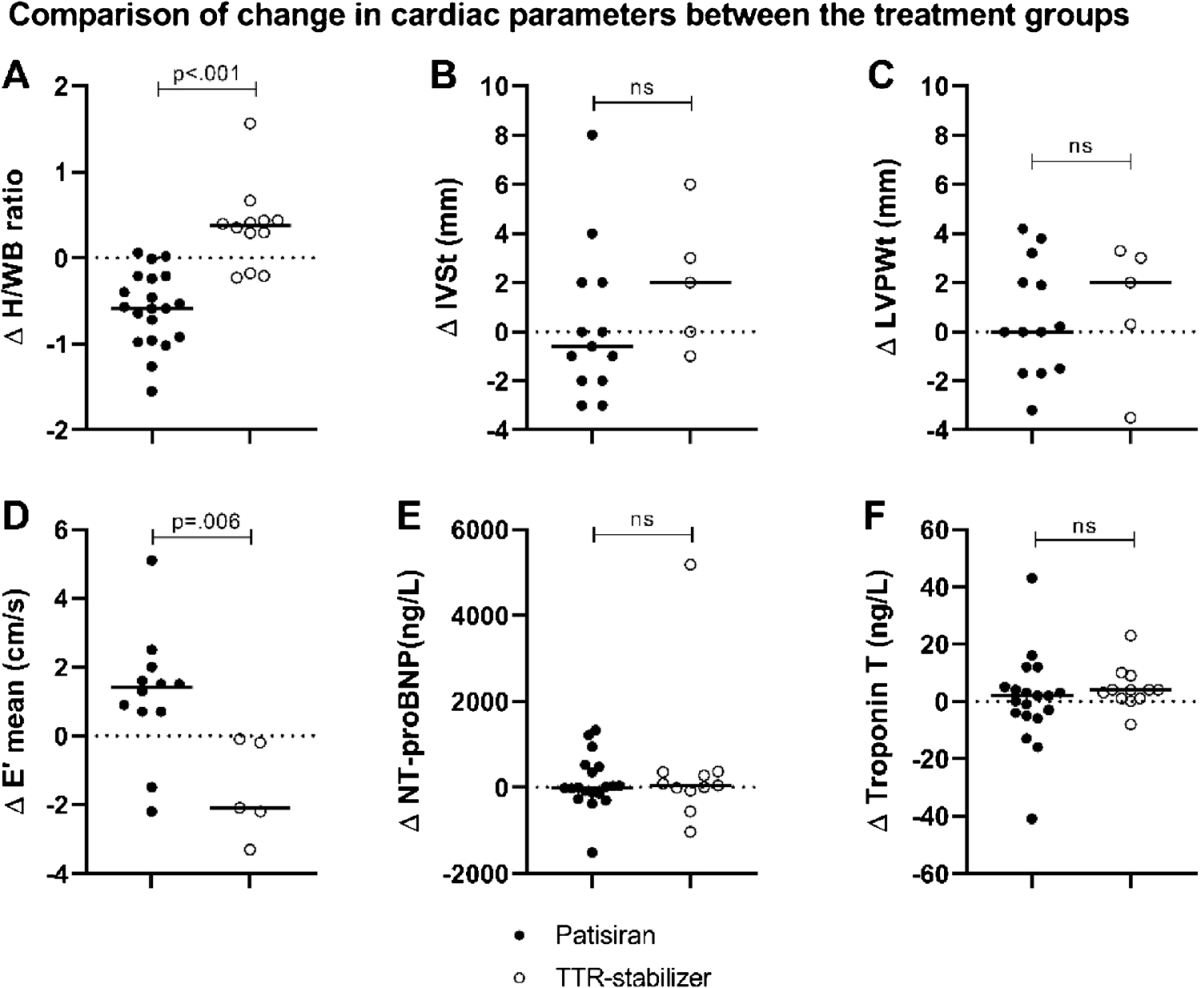
Dot-plots show the change in cardiac parameters during treatment with patisiran or a TTR-stabilizer. The solid horizontal lines represent group medians. **A** Change in H/WB ratio on bone scintigraphy. **B** Change in IVSt on echocardiography. **C** Change in LVPWt on echocardiography. **D** Change in E′ mean on echocardiography. **E** Change in NT-proBNP levels. **F** Change in troponin T levels. TTR, transthyretin; H/WB, heart/whole-body; IVSt, interventricular septal wall thickness; LVPWt, left ventricular posterior wall thickness; NT-proBNP, N-terminal brain natriuretic propeptide; ns, not significant

Change in H/WB ratio differed between the treatment groups. During treatment with patisiran, a median decrease in H/WB ratio of  − 0.58 [− 0.95 to  − 0.22] was observed, whereas an increase of  + 0.38 [− 0.06 to + 0.44] was observed in patients treated with a TTR-stabilizer (*p* < 0.001) (Fig. 4A). Additionally, the change in E′ mean differed between the groups. With an observed median change in E′ mean of  + 1.4 [+ 0.7 to  + 1.9] cm/s in the patisiran group and  − 2.1 [− 2.8 to  − 0.2] cm/s in the TTR-stabilizer group (*p* = 0.006) (Fig. 4D). No difference in change in wall thickness, E/e′ ratio, E/A ratio, and MVDT on echocardiography, NYHA class, and cardiac biomarkers was observed between the groups (Fig. 4B, C and E, F).

## Discussion

This study shows reduction of cardiac tracer uptake on bone scintigraphy in ATTRv amyloidosis patients treated with patisiran, while NYHA class, cardiac biomarkers, and parameters on echocardiography did not change. In contrast, cardiac tracer uptake on bone scintigraphy and troponin T levels increased in ATTRv amyloidosis patients treated with a TTR-stabilizer. Our results indicate that cardiac tracer uptake on bone scintigraphy is a more sensitive marker of both disease progression and treatment response as compared to the conventional follow-up parameters. Bone scintigraphy may be suitable for monitoring of ATTRv amyloidosis patients.

Our data support further exploration of bone scintigraphy in the follow-up of patients with ATTR cardiomyopathy. Our findings are in contrast with the findings of Castaño et al., who concluded that serial [^99m^Tc]Tc-pyrophosphate (PYP) bone scintigraphies could not detect disease progression in ATTR amyloidosis patients [19]. However, they studied bone scintigraphy in ATTR-CM patients with more advanced disease as compared to our study. Furthermore, more recently, we and others reported cases in which changes in cardiac tracer uptake on bone scintigraphy were observed in individual cases, case series, or patient groups [12, 20–26]. The discussion on whether bone scintigraphy can be used to monitor ATTR amyloidosis patients is ongoing, and no consensus has been reached [27, 28]. Our findings aid in resolving this discussion.

A decrease in cardiac tracer uptake on bone scintigraphy in ATTR amyloidosis patients has been associated with a decrease in extracellular volume on cardiac MRI in studies investigating the effect of patisiran [12] and antibody NI006 [26] and might therefore reflect regression of amyloid deposits. However, the binding of radionuclide-labelled phosphonates to amyloid-containing hearts depends on the presence of clouds of microcalcifications, which do not seem to be directly associated with amyloid fibrils [29, 30]. Therefore, the changes in cardiac tracer uptake likely do not directly reflect changes in amyloid load, but probably reflect a change in the calcium content of the amyloid. Whether this is part of a series of events in the regression and accumulation of amyloid needs histological confirmation.

We observed a decrease in H/WB ratio on bone scintigraphy in the absence of changes in echocardiographic parameters, which is in line with the findings of Fontana et al. in a previous study to the effect of patisiran [12]. However, analysis of the APOLLO study data did show improvement of echocardiographic parameters in 126 ATTRv amyloidosis patients with cardiomyopathy after 18 months of treatment with patisiran [11, 14]. The reproducibility of measuring wall thickness on echocardiography might not be sufficient to detect the subtle changes during a short follow-up [31]. Furthermore, patients in the current study seem to have a relatively mild cardiac phenotype, as the median NT-proBNP level is lower as compared to the patients in the aforementioned studies, which might explain the lack of improvement of echocardiographic parameters during follow-up.

We found an increase in H/WB ratio in the ATTRv amyloidosis patients treated with TTR-stabilizers, which has never been reported before. Contrarily, a decrease in cardiac tracer uptake on bone scintigraphy has been reported in wild type ATTR amyloidosis patients and one ATTRv amyloidosis patient treated with tafamidis [21–25]. The difference in outcomes between our study and previous studies might be explained by the lower dose of 20-mg tafamidis received by our patients with ATTRv amyloidosis for the indication polyneuropathy as opposed to 61 [21] or 80 mg daily [22, 23] for the indication cardiomyopathy (predominantly in wild type ATTR amyloidosis patients) in the other studies. The observed increase in H/WB ratio may therefore reflect disease progression as a result of the low dose of tafamidis as previous studies have shown that disease progresses also under treatment with tafamidis [9, 32] and as a dose of 80 mg daily has been associated with better long-term survival compared to a dose of 20 mg daily [5]. However, confirmation through histopathological studies or studies using cardiac magnetic resonance imaging is needed. Our findings on echocardiography and cardiac biomarkers in this group are in line with previous findings, as we observed no change in all echocardiographic parameters and NT-proBNP levels, but observed a slight increase in troponin T levels [33].

No differences in cardiac parameters were found between patients treated with patisiran monotherapy and patients treated with a combination of patisiran and a TTR-stabilizer, despite the potentially synergistic action of TTR-stabilizers and *TTR* gene silencers [34]. However, it needs to be stressed that groups are small. Studies specifically designed to compare combined treatment with gene silencer monotherapy are desirable.

### Limitations

The current study has several limitations. First of all, due to the retrospective design of this study, data were missing, and cardiac magnetic resonance scans (CMR) were unavailable in the majority of our patients, leaving us unable to relate bone scintigraphy findings to extracellular volume as surrogate marker of cardiac amyloid load. Also, echocardiographic GLS, which could be another early marker of treatment response [11], was not available in most of our patients.

Additionally, it would have been interesting to use SPECT quantification to measure cardiac tracer uptake on bone scintigraphy instead of a semi-quantitative ratio [35]. This was unfortunately not feasible within our retrospective cohort as quantifiable SPECT was not routinely performed at our center at the time of inclusion. SPECT quantification might allow for the detection of even more subtle changes in cardiac tracer uptake compared to semi-quantitative ratios measured on planar images [21, 24]. Therefore, our finding that a change in cardiac tracer uptake can be detected even on planar images, which are available in nearly all ATTR-CM patients, illustrates the potential for bone scintigraphy to aid in the follow-up of ATTR-CM patients even if quantitative SPECT is not available.

Finally, treatment groups were somewhat heterogeneous due to the inclusion of patients treated with tafamidis as well as diflunisal. Also, severity of cardiac phenotype differed between the groups, being milder in patients treated with a TTR-stabilizer. This could have introduced bias as patients treated with patisiran had greater “opportunity” to show improvement of cardiac parameters, whereas this was unlikely in the mild phenotype of patients treated with a TTR-stabilizer.

### Implications

The finding that bone scintigraphy might reflect treatment effect and disease progression before conventional follow-up markers do, highlights the potential of nuclear imaging in the follow-up of ATTR amyloidosis patients. However, robust quantification methods are likely to be preferred in individual follow-up. Therefore, advanced SPECT/CT quantification [21, 25, 35] and other nuclear imaging techniques such as positron emission tomography (PET) [36, 37] have to be further explored.

## Conclusion

Our findings demonstrate that bone scintigraphy might detect favorable treatment effect of patisiran in ATTRv amyloidosis patients and possible disease progression in ATTRv amyloidosis patients treated with a TTR-stabilizer at low dose. This study shows the potential of bone scintigraphy to distinguish responders from non-responders during treatment, potentially already after 1 year of follow-up.

### Supplementary Information

Below is the link to the electronic supplementary material.

Supplementary file1 (PDF 274 KB)

## Data Availability

The datasets generated during and/or analyzed during the current study are available from the corresponding author on reasonable request.

## Abbreviations

ATTR-CM: ATTR amyloid cardiomyopathy
ATTRv: Hereditary transthyretin amyloidosis
CMR: Cardiac magnetic resonance
DPD: 2,3-Dicarboxypropane-1,1-diphosphonic acid
GLS: Global longitudinal strain
H/WB: Heart-to-whole-body
HDP: Hydroxydiphosphonate
IVSt: Interventricular septal wall thickness
LVPWt: Left ventricular posterior wall thickness
MVDT: Mitral valve deceleration time
NT-proBNP: N-terminal brain natriuretic propeptide
PET: Positron emission tomography
PYP: Pyrophosphate
SPECT: Single-photon emission computed tomography
TTR: Transthyretin
