# Supplementary material for: Cardiac [99mTc]Tc-hydroxydiphosphonate uptake on bone scintigraphy in patients with hereditary transthyretin amyloidosis: an early follow-up marker?
Source: Eur J Nucl Med Mol Imaging. 2023 Oct 16;51(3):681–90. doi: 10.1007/s00259-023-06459-y (PMC10796605; doi:10.1007/s00259-023-06459-y)
Supplement: Supplementary file 1 — Supplementary file1 (PDF 274 KB) [file 259_2023_6459_MOESM1_ESM.pdf]

**Supplementary information to:**

***Title:*** Cardiac [$^{99m}$Tc]Tc-hydroxydiphosphonate uptake on bone scintigraphy in patients with hereditary transthyretin amyloidosis: an early follow-up marker?

***Journal:*** European Journal of Nuclear Medicine and Molecular Imaging

***Authors***: Tingen HSA, Tubben A, Bijzet J, van den Berg MP, van der Meer P, Houwerzijl EJ, Muntinghe FLH, van der Zwaag PA, Glaudemans AWJM, Oerlemans MIFJ, Knackstedt C, Michels M, Hirsch A, Hazenberg BPC, Slart RHJA, Nienhuis HLA

***Details corresponding author:***

Hendrea Tingen

Amyloidosis Centre of Expertise

University Medical Center Groningen

Hanzeplein 1

9713GZ Groningen

h.s.a.tingen@umcg.nl

**Online Resource 1**

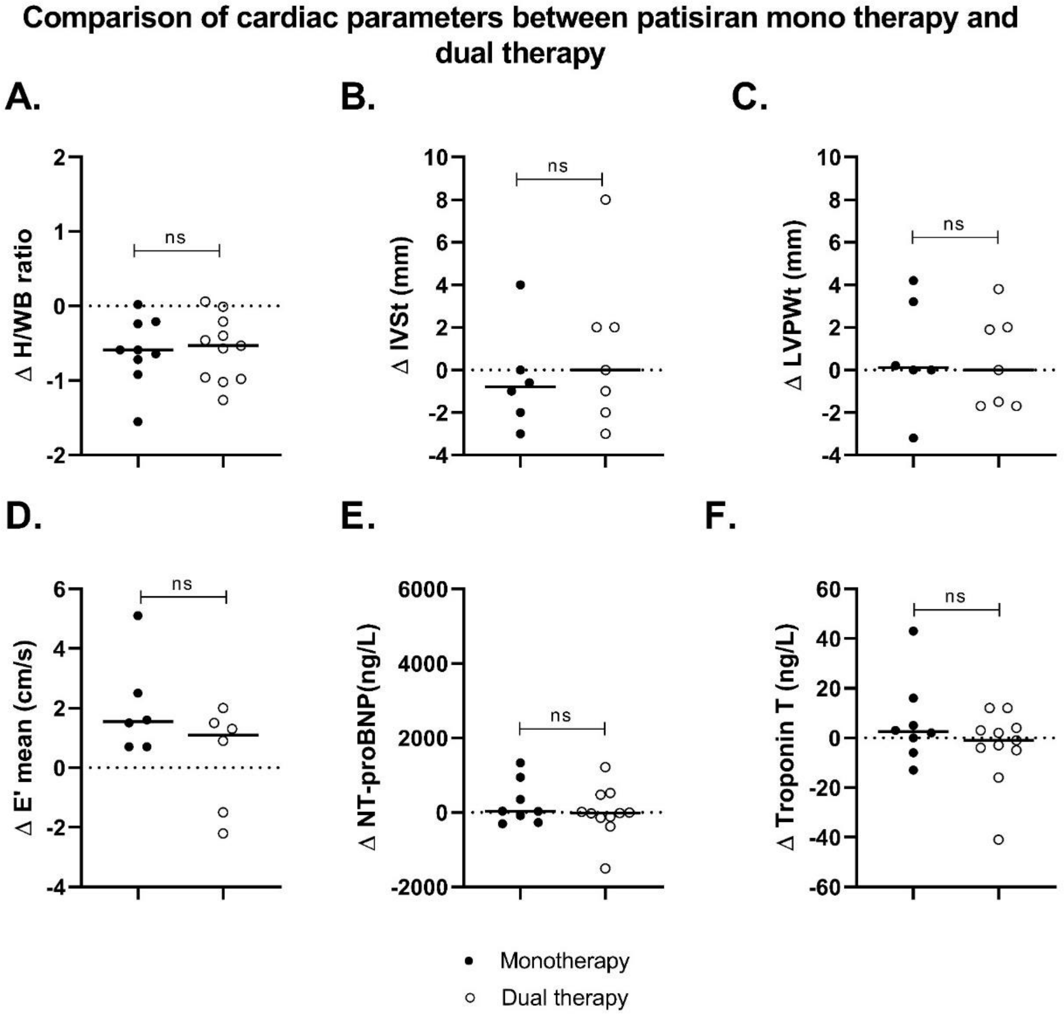


**Online Resource 1** Dot plots show the change in cardiac parameters during treatment with patisiran only or a combination of patisiran with a TTR-stabilizer. The solid horizontal lines represent group medians. A: change in H/WB ratio on bone scintigraphy. B: change in IVSt on echocardiography. C: change in LVPWt on echocardiography. D: change in E' mean on echocardiography. E: change in NT-proBNP levels. F: change in troponin T level

H/WB = heart/whole-body; IVSt = interventricular septal wall thickness; LVPWt = left ventricular posterior wall thickness; NT-proBNP = N-terminal brain natriuretic propeptide; ns = not significant.
